# Predicting early appropriate therapy for patients infected by carbapenem-resistant Gram-negative pathogens in intensive care units in Italy

**DOI:** 10.1186/s13756-024-01452-y

**Published:** 2024-08-26

**Authors:** Matteo Bassetti, Gianpaola Monti, Anne Santerre Henriksen, Christopher Longshaw

**Affiliations:** 1 Infectious Diseases Clinic, IRCCS Ospedale Policlinico San Martino, Genoa, Italy; 2 Department of Anesthesia and Intensive Care, ASST Grande Ospedale Metropolitano Niguarda, Milan, Italy; 3 Shionogi B.V., 50 Eastbourne Terrace, London, W2 6LG UK

**Keywords:** Antimicrobial resistance, Carbapenem resistance, Cefiderocol, Empiric therapy, Gram-negative bacteria, Intensive care unit, Predicted optimal early appropriate therapy, Surveillance, Susceptibility

## Abstract

**Background:**

Antibiotic resistance among Gram-negative bacteria in intensive care units (ICUs) is linked with high morbidity and mortality in patients. In this study, we estimated the therapeutic coverage of various antibiotics, focusing on cefiderocol and comparators, administered empirically against an infection of unknown origin in the ICU.

**Methods:**

In the ARTEMIS surveillance study, susceptibilities of 624 Italian Gram-negative isolates to amikacin, aztreonam-avibactam, cefiderocol, ceftazidime-avibactam, ceftolozane-tazobactam, colistin, imipenem-relebactam, meropenem, and meropenem-vaborbactam were tested by broth microdilution, and results were interpreted by European Committee on Antimicrobial Susceptibility Testing breakpoints. The susceptibility rates from the ARTEMIS study were extrapolated to Gram-negative isolates obtained from 5,774 patients in Italian ICUs in 2021. The sum of the predicted susceptibilities of individual pathogens represented the overall likelihood of in vitro activity of each antibiotic as early targeted therapy for ICU patients.

**Results:**

A total of 624 Italian Gram-negative isolates included 206 *Pseudomonas aeruginosa*, 138 *Acinetobacter baumannii*, 187 *Klebsiella pneumoniae*, and 93 *Escherichia coli*. Against *A. baumannii*, *K. pneumoniae*, *P. aeruginosa*, and *E. coli*, the overall susceptibility rates for cefiderocol were 87.7%, 96.8%, 99%, and 100%, respectively; and for comparator agents, 8.7–96.4%, 25.7–100%, 73.3–100%, and 89.2–100%, respectively. Among the subset of meropenem-resistant isolates, susceptibility rates of *A. baumannii*, *K. pneumoniae*, and *P. aeruginosa* to cefiderocol were 86.4%, 96.2% and 100%, respectively. Corresponding susceptibility rates to comparator agents were 0–96.8%, 0–100%, and 6.4–100%, respectively. There were no meropenem-resistant isolates of *E. coli*. The extrapolation of data to isolates from Italian ICUs showed that the highest likelihood of therapeutic coverage, both overall and among meropenem-resistant isolates, was reported for colistin (96.8% and 72.2%, respectively) and cefiderocol (95.7% and 71.4%, respectively). All other antibiotics were associated with a likelihood below 73% overall and between 0% and 41.4% for meropenem-resistant isolates.

**Conclusions:**

Based on confirmed susceptibility rates and reported ICU prevalence of multiple Gram-negative species, cefiderocol showed a higher predicted therapeutic coverage and utility in ICUs compared with comparator beta-lactam–beta-lactamase inhibitor antibiotics. Cefiderocol may be a promising early treatment option for patients at high risk of carbapenem-resistant Gram-negative bacterial infections in the ICU.

## Background

Gram-negative bacterial resistance is a well-known problem globally in intensive care units (ICUs) [1, 2]. A priority list of pathogens issued by the World Health Organization (WHO) in 2017 included carbapenem-resistant (CR) *Acinetobacter baumannii* (CRAB), CR Enterobacterales (CRE), and CR *Pseudomonas aeruginosa* (CRPA), for which new antibiotics are urgently needed [3]. Clinical outcomes for patients infected by these pathogens are frequently poor, and studies have identified a number of risk factors independently associated with therapeutic failure leading to death. These include the lack of effective antibiotics against difficult-to-treat resistant (DTR) strains of certain Gram-negative bacteria, the presence of host factors, mechanical ventilation, septic shock, and older age with comorbidities [2, 4, 5]. Delay in appropriate antibiotic therapy is also a risk factor. An Italian study in ICU patients with bloodstream infection (BSI) caused by CR *Klebsiella pneumoniae* carbapenemase (KPC)-producing *K. pneumoniae* showed a temporal association between mortality and appropriate antibiotic treatment over the first 72 h of management [5].

**Fig. 1:**
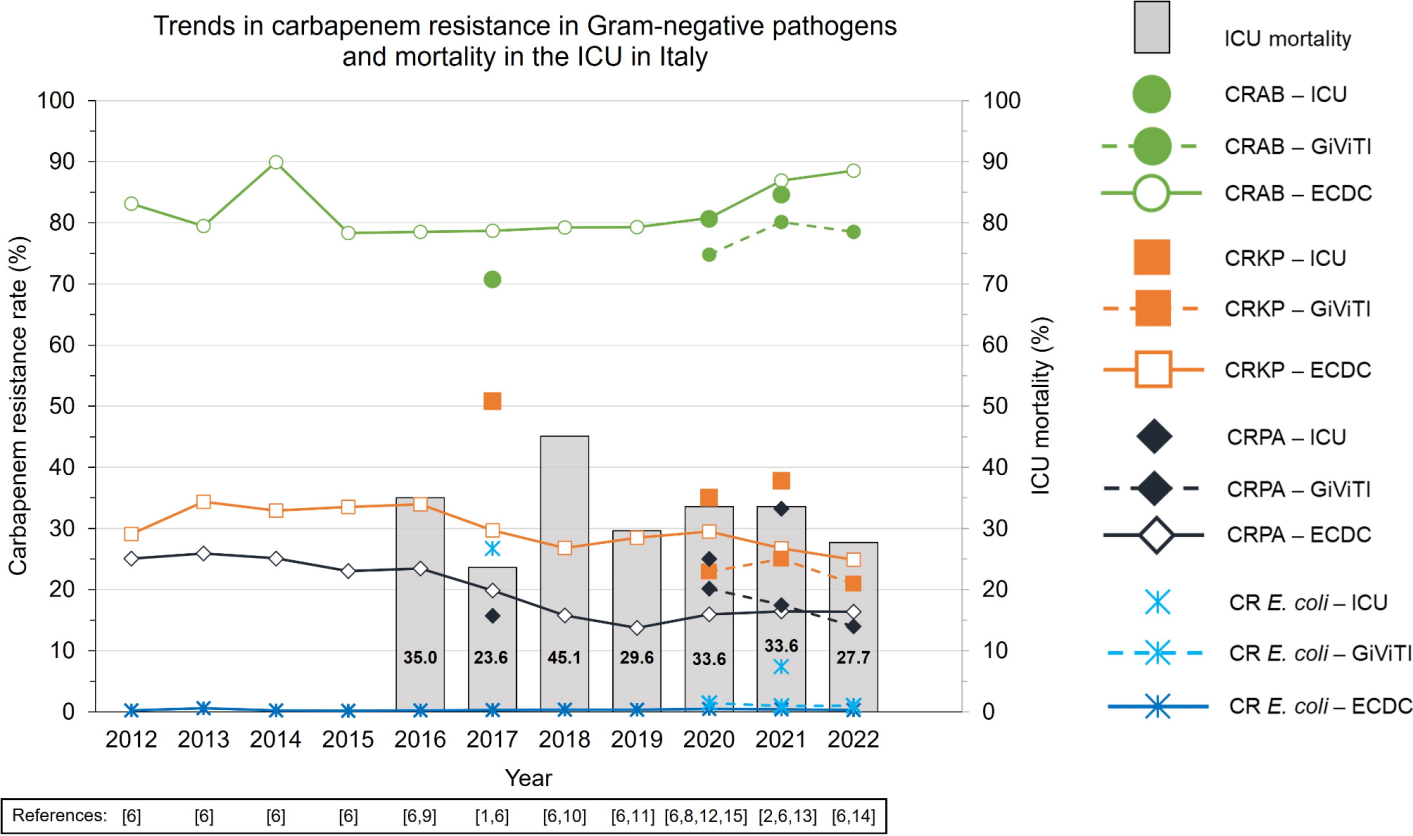
Trends in carbapenem resistance in Gram-negative pathogens and mortality in Italian ICUs. Adapted from [1, 2, 6, 9–15] Disclaimers **ECDC** [6]: Dataset provided by ECDC based on data provided by WHO and Ministries of Health from the affected countries. The views and opinions of the authors expressed herein do not necessarily state or reflect those of the ECDC. The accuracy of the authors’ statistical analysis and the findings they report are not the responsibility of ECDC. ECDC is not responsible for conclusions or opinions drawn from the data provided. ECDC is not responsible for the correctness of the data and for data management, data merging, and data collation after provision of the data. ECDC shall not be held liable for improper or incorrect use of the data **GiViTI** [12–14]: Data are adapted from annual reports by Gruppo Italiano per la Valutazione degli Interventi in Terapia Intensiva CR, carbapenem-resistant; CRAB, carbapenem-resistant *Acinetobacter baumannii*; CRKP, carbapenem-resistant *Klebsiella pneumoniae*; CRPA, carbapenem-resistant *Pseudomonas aeruginosa*; ECDC, European Centre for Disease Prevention and Control; *E. coli*, *Escherichia coli*; GiViTI, Gruppo Italiano per la Valutazione Degli Interventi in Terapia Intensiva; ICU, intensive care unit; WHO, World Health Organization.

In a recent international cohort of patients with ICU-acquired BSIs, carbapenem resistance was the greatest among *Acinetobacter* spp. (84.6%) and was also reported in one-third of *K. pneumoniae* and *P. aeruginosa* isolates [2]. Additionally, a large proportion of isolates were detected as DTR or pan-drug resistant (PDR) strains [2]. According to data from the European Centre for Disease Prevention and Control (ECDC), annual carbapenem resistance between 2012 and 2022 in Italy was approximately 80–90% in *Acinetobacter* spp., 25–34% in *K. pneumoniae*, 14–26% in *P. aeruginosa*, and below 1% in *Escherichia coli* (Fig. 1) [6]. However, in some ICUs, carbapenem resistance may exceed nationally reported percentages (Fig. 1) [7–15].

It is likely that antimicrobial resistance in ICUs may have been exacerbated by the coronavirus disease 2019 (COVID-19) pandemic. In the initial phase of the pandemic, several factors, including increased antibiotic use, lack of appropriate infection control, and rapid transmission of multidrug-resistant (MDR) Gram-negative pathogens in ICUs, were reported, all of which contributed to the increase in antimicrobial resistance both in Italy and globally [16–19]. Since then, Gram-negative bacterial resistance in Italian ICUs, as evidenced by higher rates of CR *K. pneumoniae* in the ICU compared with other hospital departments, has persisted [20]. The COVID-19 pandemic also saw CRAB become significantly more problematic in ICUs across Italy and other European countries [21, 22]. Data from the Gruppo Italiano per la Valutazione degli Interventi in Terapia Intensiva (GiViTI), which indicated high levels of carbapenem resistance in Italian ICUs, highlighted the need to raise physicians’ awareness of this issue at a local level (Fig. 1) [12–14].

The impact of COVID-19 on patient outcomes was particularly significant for Italian ICUs at the outset of the pandemic, with dramatically increased mortality rates among patients with hospital-acquired infections (HAIs) [23–25]. Understanding local epidemiology and antibiotic resistance are pivotal to initiating early appropriate treatment for ICU patients infected by CR Gram-negative pathogens [26, 27]. Data from an Italian cohort of mechanically ventilated ICU patients with COVID-19 reported that 60% of HAIs were due to MDR Gram-negative bacteria [19]. Furthermore, studies have demonstrated a relationship between COVID-19 and the risk of ICU-acquired MDR bacterial infections, as well as between ICU-acquired BSI or ventilator-associated pneumonia (VAP) due to MDR pathogens and mortality [28, 29].

There is an urgent need for more effective management of CR and MDR Gram-negative bacterial infections. Cefiderocol is a parenteral siderophore cephalosporin developed for the treatment of infections caused by susceptible Gram-negative bacteria, including CRAB, CRPA, CRE, *and Stenotrophomonas maltophilia* [30]. Global surveillance studies have shown that cefiderocol has high in vitro activity against a range of aerobic Gram-negative bacteria with different phenotypic profiles [31–33].

The objectives of the current study were to compare the in vitro activity of cefiderocol with that of comparator antibiotics against Gram-negative bacteria collected in Italy in 2020, and to predict the therapeutic coverage and potential utility of cefiderocol for the early treatment of ICU patients at high risk of CR-Gram-negative bacterial infection.

## Methods

This study examined the likelihood of therapeutic coverage of antibiotic treatments (overall and among CR Gram-negative isolates) against a suspected bacterial infection of unconfirmed aetiology or susceptibility, based on the prevalence of Gram-negative isolates in Italian ICUs and tested susceptibility from the Italian cohort of the ARTEMIS surveillance study.

### Surveillance study

The ARTEMIS surveillance study was conducted by a central laboratory (IHMA Sarl, Monthey, Switzerland) to evaluate the in vitro activity of cefiderocol and comparator antibiotics against Gram-negative isolates (Enterobacterales, *P. aeruginosa*, and *Acinetobacter* spp.). Isolates were collected from hospitalised patients with Gram-negative bacterial infections across 49 centres in France, Germany, Austria, Spain, Italy, and the United Kingdom (UK) between 1 January and 31 December 2020; patients with complicated urinary tract infection (UTI) were excluded. Details about the surveillance programme are provided elsewhere [34, 35]. In the current study, data for isolates of Enterobacterales, *P. aeruginosa*, and *A. baumannii* from hospitals in Italy were assessed.

### Susceptibility testing

Susceptibility testing was performed according to Clinical and Laboratory Standards Institute (CLSI) guidelines [36]. In brief, minimum inhibitory concentration (MIC) testing was conducted on 96-well broth microdilution microtitre plates prepared in-house, with the exception of ceftazidime-avibactam, testing for which was performed using Sensititre freeze-dried panels (Thermo Fisher, Reinach, Switzerland). 50 µL of antibiotic solutions at 2x of the final concentrations in cation-adjusted Mueller-Hinton broth (CAMHB), and in iron-depleted cation-adjusted Mueller-Hinton broth (ID-CAMHB) for cefiderocol, were added into 96-well microtitre plates. Microtitre plates were stored frozen at − 80 °C until the day of test for a maximum of 6 months and never thawed more than once. Bacterial inocula were prepared at approximately 1 × 10^6^ CFU/mL by diluting 100-fold a 0.5 McFarland suspension in CAMHB or ID-CAMHB. Antibacterial microtitre plate wells previously filled with 50 µL of antibiotic solutions were diluted 2-fold with 50 µL of bacterial inoculum to reach a final density of 5 × 10^5^ CFU/mL and the final test concentrations of antibiotics. For ceftazidime-avibactam susceptibility testing, 100 µL of bacterial inoculum was added to the Sensititre panel to reach a final density of 5 × 10^5^ CFU/mL.

MIC evaluations were performed several times (i.e., up to five times for discordant results); if differing results were obtained, the geometric mean rounded to the closest MIC was used. The following CLSI quality control strains were also included on each day of testing: *E. coli* ATCC 25922, *P. aeruginosa* ATCC 27853, *A. baumannii* ATCC 13304, and *K. pneumoniae* ATCC 700603.

In the current analysis, the comparator antimicrobials included amikacin, colistin, ceftazidime-avibactam, ceftolozane-tazobactam, imipenem-relebactam, meropenem, meropenem-vaborbactam, and aztreonam-avibactam. These were prepared according to CLSI testing standards [37]. The sources of the antibiotics were as follows: amikacin, aztreonam, ceftolozane, imipenem, meropenem from USP, avibactam from Biochempartner, cefiderocol from Shionogi & Co., Ltd., ceftazidime from TOKU-E, colistin sulfate from Adooq Bioscience, relebactam from MedChemTronica, tazobactam from Selleckchem, and vaborbactam from MedChem.

Isolates were designated as susceptible or resistant to cefiderocol and comparator antibiotics according to European Committee on Antimicrobial Susceptibility Testing (EUCAST; v12) breakpoints [38]. In vitro activity of cefiderocol and comparator antibiotics were also studied against subsets of isolates according to their susceptibility phenotypes, including isolates not susceptible to either amikacin or aztreonam-avibactam, ceftazidime-avibactam, ceftolozane-tazobactam, imipenem-relebactam, meropenem, and meropenem-vaborbactam.

### Predicted therapeutic coverage in ICUs

The likelihood of early appropriate therapy and therapeutic coverage was predicted for cefiderocol and comparator antibiotics according to an approach that has been described and reported previously [39]. The prevalence of Gram-negative bacterial isolates collected and reported for Italian ICUs in 2021 [20] were cross-referenced with the susceptibility data reported in the current study. The pool of 5,774 ICU Gram-negative isolates comprised *K. pneumoniae* (33.3%), *E. coli* (24.0%), *Acinetobacter* spp. (23.6%), and *P. aeruginosa* (19.0%), collected in Italian ICUs during 2021 by the Italian National Institute of Health (Instituto Superiore di Sanita [ISS]) [20]. The susceptibilities of *K. pneumoniae*, *E. coli*, *A. baumannii*, and *P. aeruginosa* obtained from the ARTEMIS programme were used to infer the proportion of 5,774 ICU isolates likely to be susceptible (S) to cefiderocol and comparator antibiotics (S_predicted_ = S_ARTEMIS_ x n_species_/N_total_). The proportions of predicted susceptibility per species (S1, S2, S3, S4) were summed up to calculate the overall likelihood of in vitro activity for individual antibiotics (S1 + S2 + S3 + S4 = S_overall_).

## Results

### Isolates in the ARTEMIS study

**Fig. 2:**
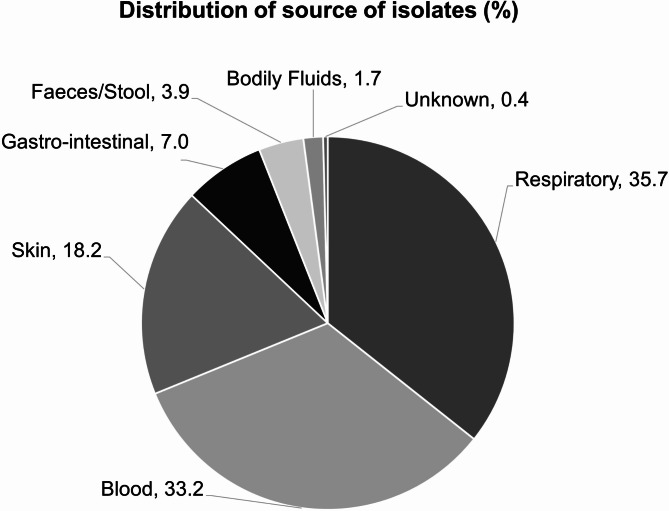
Infection sources for 771 Italian isolates^a, b^ collected in the ARTEMIS surveillance study ^a^Gram-negative isolates included in the current surveillance: *A. baumannii* 138; *P. aeruginosa* 206; *K. pneumoniae* 187; *E. coli* 93 ^b^Additional Gram-negative isolates collected in Italy: *Klebsiella oxytoca* 15; *Klebsiella aerogenes* 11; *Klebsiella variicola* 5; *Klebsiella unspeciated* 3; *Enterobacter* spp. 57; *Serratia* spp. 12; *Citrobacter* spp. 7; *Proteus* spp. 12; *Morganella morganii* 11; *Providencia stuartii* 2; *Acinetobacter* spp. 1

From January to December 2020, 771 Gram-negative isolates were collected from 11 hospitals across Italy in the ARTEMIS study, including 206 *P. aeruginosa*, 138 *A. baumannii*, 187 *K. pneumoniae*, and 93 *E. coli* isolates. The most frequent sites of biospecimens were the respiratory tract (35.7%), blood (33.2%), skin/wound (18.2%), and gastro-intestinal tract (7.0%) (Fig. 2).

### Cefiderocol MIC distribution in the ARTEMIS study

MIC distributions showed that cefiderocol MIC values were generally ≤ 2 µg/mL (EUCAST susceptibility breakpoint), except for 17 *A. baumannii*, two *P. aeruginosa*, and six *K. pneumoniae*, which had MICs of 4 µg/mL and above (Table 1). The most frequent cefiderocol MIC values for each species were: 0.5 µg/mL for *A. baumannii* complex, 0.25 µg/mL for *P. aeruginosa*, 1 µg/mL for *K. pneumoniae* (except for ceftazidime-avibactam-resistant isolates, i.e., 2 µg/mL), and 0.25 µg/mL for *E. coli*. MIC_50_ and MIC_90_ values overall and for isolates with various antibiotic resistance phenotypes are included in Table 1.

**Table 1:** Cefiderocol MIC distribution of pathogens collected in the ARTEMIS surveillance study^a, b^

	Number of isolates at MIC (µg/mL)												MIC_50_ (µg/mL)	MIC_90_ (µg/mL)
	≤ 0.03	0.06	0.12	0.25	0.5	1	2	4	8	16	32	≥ 64		
**Acinetobacter baumannii**														
*Acinetobacter baumannii*, *n* = 138	2	1	4	22	**50**	18	24	*5*	*2*	*7*		*3*	0.5	*4*
meropenem-resistant *Acinetobacter baumannii*, *n* = 125	2		1	20	**45**	17	23	*5*	*2*	*7*		*3*	0.5	*4*
amikacin-resistant *Acinetobacter baumannii*, *n* = 120	2		1	19	**42**	17	22	*5*	*2*	*7*		*3*	0.5	*4*
imipenem-relebactam-resistant *Acinetobacter baumannii*, *n* = 125	2		1	20	**45**	17	24	*5*	*2*	*6*		*3*	0.5	*4*
meropenem-vaborbactam-resistant *Acinetobacter baumannii*, *n* = 124	2		1	19	**45**	17	23	*5*	*2*	*7*		*3*	0.5	*4*
***Pseudomonas aeruginosa***														
*Pseudomonas aeruginosa*, *n* = 206	23	23	28	**64**	45	13	8		*1*	*1*			0.25	1
meropenem-resistant *Pseudomonas aeruginosa*, *n* = 47	2	8	4	**24**	8		1						0.25	0.5
amikacin-resistant *Pseudomonas aeruginosa*, *n* = 30	2	4	4	**13**	7								0.25	0.5
aztreonam-avibactam-resistant *Pseudomonas aeruginosa*, *n* = 38	2	4	4	**19**	5	2	1		*1*				0.25	1
ceftolozane-tazobactam-resistant *Pseudomonas aeruginosa*, *n* = 41	3	3	6	**16**	8	2	1		*1*	*1*			0.25	1
ceftazidime-avibactam-resistant *Pseudomonas aeruginosa*, *n* = 39	2	5	5	**15**	9		1		*1*	*1*			0.25	0.5
imipenem-relebactam-resistant *Pseudomonas aeruginosa*, *n* = 55	2	6	8	**24**	12	2	1						0.25	0.5
meropenem-vaborbactam-resistant *Pseudomonas aeruginosa*, *n* = 44	2	7	4	**23**	7		1						0.25	0.5
***Klebsiella pneumoniae***														
*Klebsiella pneumoniae*, *n* = 187	6	4	6	28	38	**55**	44	*3*	*3*				1	2
meropenem-resistant *Klebsiella pneumoniae*, *n* = 104			1	7	23	**37**	32	*2*	*2*				1	2
ceftolozane-tazobactam-resistant *Klebsiella pneumoniae*, *n* = 139		2	1	14	31	**44**	41	*3*	*3*				1	2
ceftazidime-avibactam-resistant *Klebsiella pneumoniae*, *n* = 24		1	1	1	4	4	**9**	*1*	*3*				2	*8*
***Escherichia coli***														
*Escherichia coli*, *n* = 93	8	7	15	**20**	17	17	9						0.25	1

^a^Numbers in italics indicate MICs above the EUCAST breakpoint for cefiderocol [38]

^b^Bold values represent the most frequent MIC values

EUCAST, European Committee on Antimicrobial Susceptibility Testing; MIC, minimum inhibitory concentration

Among 125 *A. baumannii* complex, 47 *P. aeruginosa*, and 104 *K. pneumoniae* isolates resistant to meropenem, cefiderocol MICs of 4 µg/mL and above were seen in 17 *A. baumannii* complex and 4 *K. pneumoniae* isolates (Table 1). For meropenem-resistant isolates, the most frequent cefiderocol MIC values were consistent with the aforementioned species-specific MIC values for all isolates.

### Antibiotic susceptibility in the ARTEMIS study

Cefiderocol showed some of the highest susceptibility rates among the antibiotics tested (Table 2), with overall susceptibilities of 87.7% for *A. baumannii* complex, 96.8% for *K. pneumoniae*, 99% for *P. aeruginosa*, and 100% for *E. coli*. Susceptibility rates of isolates to comparator agents varied: between 8.7% (imipenem-relebactam) and 96.4% (colistin) for *A. baumannii*; between 73.3% (imipenem-relebactam) and 100% (colistin) for *P. aeruginosa*; between 25.7% (ceftolozane-tazobactam) and 100% (aztreonam-avibactam) for *K. pneumoniae*; and between 89.2% (ceftazidime-avibactam) and 100% (aztreonam-avibactam) for *E. coli*.

**Table 2:** Susceptibilities (EUCAST^a^) of isolated pathogens collected in the ARTEMIS surveillance study

	MIC_50_ (µg/mL)	MIC_90_ (µg/mL)	MIC range (µg/mL)	% S	% *R*
**Overall set**					
***Acinetobacter baumannii*** **complex (** **N** ** = 138)**					
Amikacin	> 128	> 128	2–>128	13	87
*Cefiderocol*	*0.5*	*4*	*≤ 0.03*–*>32*	*87.7*	*12.3*
Colistin	≤ 0.25	0.5	≤ 0.25–>8	96.4	3.6
Imipenem-relebactam	> 16	> 16	0.25–>16	8.7	91.3
Meropenem	> 16	> 16	≤ 0.25–>16	9.4	90.6
Meropenem-vaborbactam	> 32	> 32	0.12–>32	10.1	89.9
***P. aeruginosa *** **(** ***N*** ** = 206)**					
Amikacin	4	64	1–>128	85.4	14.6
Aztreonam-avibactam	8	32	≤ 1–>32	81.6	18.4
*Cefiderocol*	*0.25*	*1*	*≤ 0.03*–*16*	*99*	*1*
Ceftazidime-avibactam	2	> 16	≤ 0.25–>16	81.1	18.9
Ceftolozane-tazobactam	1	> 32	0.5–>32	80.1	19.9
Colistin	1	1	≤ 0.25–4	100	0
Imipenem-relebactam	0.5	> 16	≤ 0.12–>16	73.3	26.7
Meropenem	1	> 16	≤ 0.25–>16	77.2	22.8
Meropenem-vaborbactam	1	> 32	≤ 0.06–>32	78.6	21.4
***K. pneumoniae *** **(** ***N*** ** = 187)**					
Amikacin	4	> 128	≤ 0.5–>128	55.6	44.4
Aztreonam-avibactam	≤ 1	≤ 1	≤ 1–2	100	0
*Cefiderocol*	*1*	*2*	*≤ 0.03*–*8*	*96.8*	*3.2*
Ceftazidime-avibactam	1	> 16	≤ 0.25–>16	87.2	12.8
Ceftolozane-tazobactam	> 32	> 32	0.25–>32	25.7	74.3
Colistin	≤ 0.25	≤ 0.25	≤ 0.25–>8	94.7	5.3
Imipenem-relebactam	0.25	2	≤ 0.12–>16	90.9	9.1
Meropenem	> 16	> 16	≤ 0.25–>16	44.4	55.6
Meropenem-vaborbactam	0.12	4	≤ 0.06–>32	93.6	6.4
***E. coli *** **(** ***N*** ** = 93)**					
Amikacin	4	8	1–128	96.8	3.2
Aztreonam-avibactam	≤ 1	≤ 1	≤ 1–≤1	100	0
*Cefiderocol*	*0.25*	*1*	*≤ 0.03*–*2*	*100*	*0*
Ceftazidime-avibactam	≤ 0.25	16	≤ 0.25–>16	89.2	10.8
Ceftolozane-tazobactam	0.5	2	≤ 0.12–>32	91.4	8.6
Colistin	≤ 0.25	≤ 0.25	≤ 0.25–>8	97.8	2.2
Imipenem-relebactam	≤ 0.12	0.25	≤ 0.12–16	96.8	3.2
Meropenem	≤ 0.25	≤ 0.25	≤ 0.25–16	98.9	1.1
Meropenem-vaborbactam	≤ 0.06	≤ 0.06	≤ 0.06–32	98.9	1.1
**Meropenem-resistant subset**					
***Acinetobacter baumannii*** **complex (** ***N*** ** = 125)**					
Amikacin	> 128	> 128	2–>128	4.8	95.2
*Cefiderocol*	*0.5*	*4*	*≤ 0.03*–>32	*86.4*	*13.6*
Colistin	≤ 0.25	0.5	≤ 0.25–>8	96.8	3.2
Imipenem-relebactam	> 16	> 16	4–>16	0	100
Meropenem	> 16	> 16	16–>16	0	100
Meropenem-vaborbactam	> 32	> 32	8–>32	0.8	99.2
***P. aeruginosa *** **(** ***N*** ** = 47)**					
Amikacin	16	128	2–>128	53.2	46.8
Aztreonam-avibactam	32	> 32	8–>32	42.6	57.4
*Cefiderocol*	*0.25*	*0.5*	*≤ 0.03*–*2*	*100*	*0*
Ceftazidime-avibactam	16	> 16	1–>16	42.6	57.4
Ceftolozane-tazobactam	8	> 32	1–>32	44.7	55.3
Colistin	1	1	0.5–1	100	0
Imipenem-relebactam	8	> 16	1–>16	6.4	93.6
Meropenem	> 16	> 16	16–>16	0	100
Meropenem-vaborbactam	32	> 32	8–>32	6.4	93.6
***K. pneumoniae *** **(** ***N*** ** = 104)**					
Amikacin	32	> 128	≤ 0.5–>128	32.7	67.3
Aztreonam-avibactam	≤ 1	≤ 1	≤ 1–2	100	0
*Cefiderocol*	*1*	*2*	*0.12*–*8*	*96.2*	*3.8*
Ceftazidime-avibactam	2	> 16	≤ 0.25–>16	87.5	12.5
Ceftolozane-tazobactam	> 32	> 32	32–>32	0	100
Colistin	≤ 0.25	1	≤ 0.25–>8	91.3	8.7
Imipenem-relebactam	0.5	4	≤ 0.12–>16	88.5	11.5
Meropenem	> 16	> 16	16–>16	0	100
Meropenem-vaborbactam	1	16	≤ 0.06–>32	89.4	10.6

^a^EUCAST breakpoints were defined according to [38]. EUCAST high-dose meropenem breakpoint was used to identify meropenem-resistant isolates [38]

EUCAST, European Committee on Antimicrobial Susceptibility Testing; MIC, minimum inhibitory concentration; R, resistant; S, susceptible

Among the subset of meropenem-resistant isolates (Table 2), susceptibilities to cefiderocol were 86.4% for *A. baumannii* complex, 100% for *P. aeruginosa*, and 96.2% for *K. pneumoniae*. There were no meropenem-resistant *E. coli* isolates in the study. Susceptibility rates to comparator agents ranged between 0% (imipenem-relebactam) and 96.8% (colistin) for *A. baumannii*; between 6.4% (imipenem-relebactam, meropenem-vaborbactam) and 100% (colistin) for *P. aeruginosa*; and between 0% (ceftolozane-tazobactam) and 100% (aztreonam-avibactam) for *K. pneumoniae*.

### Predicted therapeutic coverage in ICUs

Table 3 shows the predicted susceptibilities of different antibiotics based on the comparative breadth of in vitro coverage against the most frequent resistant pathogen profiles. The proportions of *Acinetobacter* spp., *P. aeruginosa*, *K. pneumoniae*, and *E. coli* were reported to be similar across ICUs (Table 3). The highest likelihood of susceptibility, both overall and among meropenem-resistant isolates, was reported for colistin (96.8% and 72.2%, respectively) and cefiderocol (95.7% and 71.4%, respectively). All other antibiotics were associated with a likelihood below 73% overall and between 0% and 41.4% among meropenem-resistant isolates.

**Table 3:** Susceptibility^a^ and predicted therapeutic coverage and utility against Gram-negative bacterial isolates in Italian ICUs

Overall	Total isolates	Pathogen			
		*Acinetobacter spp.*	*P*. aeruginosa	*K. pneumoniae*	*E. coli*
**ICU**, **N**^**b**^	5,774	1363	1099	1924	1388
**ICU**, **%**	100	23.6	19.0	33.3	24.0
**Amikacin**					
Susceptible (%) – overall	NA	13.0	85.4	55.6	96.8
Predicted coverage^c^ (%) – overall	**61.0**	3.1	16.2	18.5	23.2
Susceptible (%) – meropenem resistant	NA	4.8	53.2	32.7	NA
Predicted coverage^c^ (%) – meropenem resistant	**22.1**	1.1	10.1	10.9	NA
**Aztreonam-avibactam**					
Susceptible (%) – overall	NA	NS^d^	81.6	100	100
Predicted coverage^c^ (%) – overall	**72.8**	0	15.5	33.3	24.0
Susceptible (%) – meropenem resistant	NA	NS^d^	42.6	100	NA
Predicted coverage^c^ (%) – meropenem resistant	**41.4**	0	8.1	33.3	NA
**Cefiderocol**					
Susceptible (%) – overall	NA	87.7	99	96.8	100
Predicted coverage^c^ (%) – overall	**95.7**	20.7	18.8	32.2	24.0
Susceptible (%) – meropenem resistant	NA	86.4	100	96.2	NA
Predicted coverage^c^ (%) – meropenem resistant	**71.4**	20.4	19.0	32.0	NA
**Ceftazidime-avibactam**					
Susceptible (%) – overall	NA	NS^d^	81.1	87.2	89.2
Predicted coverage^c^ (%) – overall	**65.8**	0	15.4	29.0	21.4
Susceptible (%) – meropenem resistant	NA	NS^d^	42.6	87.5	NA
Predicted coverage^c^ (%) – meropenem resistant	**37.2**	0	8.1	29.1	NA
**Ceftolozane-tazobactam**					
Susceptible (%) – overall	NA	NS^d^	80.1	25.7	91.4
Predicted coverage^c^ (%) – overall	**45.7**	0	15.2	8.6	21.9
Susceptible (%) – meropenem resistant	NA	NS^d^	44.7	0	NA
Predicted coverage^c^ (%) – meropenem resistant	**8.5**	0	8.5	0	NA
**Colistin**					
Susceptible (%) – overall	NA	96.4	100	94.7	97.8
Predicted coverage^c^ (%) – overall	**96.8**	22.8	19.0	31.5	23.5
Susceptible (%) – meropenem resistant	NA	96.8	100	91.3	NA
Predicted coverage^c^ (%) – meropenem resistant	**72.2**	22.8	19.0	30.4	NA
**Imipenem-relebactam**					
Susceptible (%) – overall	NA	8.7	73.3	90.9	96.8
Predicted coverage^c^ (%) – overall	**69.5**	2.1	13.9	30.3	23.2
Susceptible (%) – meropenem resistant	NA	0	6.4	88.5	NA
Predicted coverage^c^ (%) – meropenem resistant	**30.7**	0	1.2	29.5	NA
**Meropenem**					
Susceptible (%) – overall	NA	9.4	77.2	44.4	98.9
Predicted coverage^c^ (%) – overall	**55.4**	2.2	14.7	14.8	23.7
Susceptible (%) – meropenem resistant	NA	0	0	0	NA
Predicted coverage^c^ (%) – meropenem resistant	**0**	0	0	0	NA
**Meropenem-vaborbactam**					
Susceptible (%) – overall	NA	10.1	78.6	93.6	98.9
Predicted coverage^c^ (%) – overall	**72.2**	2.4	14.9	31.2	23.7
Susceptible (%) – meropenem resistant	NA	0.8	6.4	89.4	NA
Predicted coverage^c^ (%) – meropenem resistant	**31.2**	0.2	1.2	29.8	NA

^a^Actual susceptibility obtained in the ARTEMIS surveillance study was based on EUCAST breakpoints [38]

^b^The total number of isolates from Italian ICUs was based on reported percentages [Adapted from 20]

^c^Predicted coverage or susceptibility (%) is the likelihood that the selected antibiotic would be appropriate in the ICU when the species and its susceptibility are not known. The actual susceptibilities of *K. pneumoniae*, *E. coli*, *A. baumannii*, and *P. aeruginosa* (data in Table 2) obtained from the ARTEMIS programme were used to infer the proportion of 5,774 ICU isolates likely to be susceptible (S) to cefiderocol and comparator antibiotics (S_predicted_ = S_ARTEMIS_ x n_species_/N_total_). The proportions of predicted coverage per species (S1, S2, S3, S4) were summed up to calculate the overall likelihood of in vitro activity and therapeutic coverage for individual antibiotics (S1 + S2 + S3 + S4 = S_overall_)

^d^*Acinetobacter baumannii* was not considered susceptible to aztreonam-avibactam, ceftazidime-avibactam, and ceftolozane-tazobactam

EUCAST, European Committee on Antimicrobial Susceptibility Testing; ICU, intensive care unit; NA, not applicable; NS, not susceptible

## Discussion

In the current study, we compared the likelihood of therapeutic coverage of various antibiotics – including the newer agents: cefiderocol, ceftazidime-avibactam, meropenem-vaborbactam, imipenem-relebactam and aztreonam-avibactam – against meropenem-resistant strains of Gram-negative pathogens frequently encountered in ICUs in Italy (Fig. 1). ICU patients with CR Gram-negative bacterial infections often have limited empiric treatment options because of a combination of factors, such as different antibiotic coverage and unknown susceptibility profiles of pathogens. Among the antibiotics evaluated in this study, colistin, a polymyxin antibiotic, and cefiderocol, a beta-lactam antibiotic, were the two antibiotics most likely to be active against Gram-negative bacterial isolates obtained in the ICU in the absence of culture results.

ICU epidemiological data reported VAP, catheter-related BSI, and catheter-related UTI as the most frequent diagnoses in Italian ICU patients [12–14, 19, 40]. This is broadly in line with the data in our study on the origin of isolates. Italian national reports have suggested that mortality rates among ICU patients with HAIs or ICU-acquired infections vary by infection severity, diagnosis, and COVID-19 status. In 2022, a mortality rate of 10–13% was reported for infected ICU patients without sepsis, 18–28% for infected septic patients, and ∼ 50% for infected patients with septic shock. Mortality rates were twice as high among patients with COVID-19 pneumonia versus those without [12–14, 28, 29, 40].

The efficacy and safety of the newer beta-lactam and beta-lactam–beta-lactamase inhibitor antibiotics against CRE and CRAB were investigated in several pathogen-focused randomised clinical studies, using the best available therapy – including colistin or tigecycline – as comparator arms [41–44]. Although these studies had their own limitations, such as the administration of newer agents in monotherapy and small patient sample sizes due to the relatively low global prevalence of CR Gram-negative pathogens [45], they led to the introduction of agents – including ceftazidime-avibactam, meropenem-vaborbactam, imipenem-relebactam and cefiderocol – for the treatment of infections caused by CR Gram-negative bacteria; all of which have demonstrated clinical efficacy in real-world settings [21, 46–49]. Current clinical practice guidelines (European Respiratory Society 2018) for the treatment of patients with VAP and hospital-acquired pneumonia (HAP) recommend stratifying patients by the potential risk of being infected by MDR pathogens, such as CRPA or CRAB, as well as the presence of sepsis and septic shock [50]. Patients with prior treatment failure in the highest risk groups may benefit from more individualised therapy, which could include the addition of polymyxin or tigecycline [50]. However, antibiotics with a more favourable safety profile – such as cefiderocol, ceftazidime-avibactam, and imipenem-relebactam – may be preferred over antibiotics that are known to be associated with toxicity [51, 52]. Hospitalised patients with HAP or VAP may be infected or colonised by different MDR and CR Gram-negative pathogens with a variety of resistance mechanisms. These warrant the need for empiric antibiotic treatments with the versatility to cover a range of pathogens and mechanisms of resistance [26]. The Italian guidelines for patients with serious VAP recommend treatment with the newer beta-lactam–beta-lactamase inhibitors or cefiderocol for CR, MDR, and extensively drug-resistant (XDR) Gram-negative bacterial infections, with agent selection based on the mechanism of resistance [53].

A recent consensus document by an Italian multidisciplinary team addressing antimicrobial stewardship as best practice for critically ill patients in the ICU highlighted that the timing of appropriate antibiotic treatment is crucial to minimising morbidity and mortality [54]. The consensus statement placed an emphasis on rapid microbiological diagnosis, local antibiogram, and surveillance data to better guide antibiotic therapy [54]. According to a recent review of rapid diagnostic methods for identifying molecular mechanisms in causative pathogens, antimicrobial stewardship practices can be strengthened by shortening the time to appropriate therapy, with beneficial impact on clinical outcomes [55]. This is supported by another study by Rivera-Villegas et al., in which appropriate antibiogram-guided antibiotic treatment was associated with better outcomes in patients with CR Gram-negative bacterial infections [56]. On the other hand, inappropriate empiric antibiotic treatment in patients with CR *P. aeruginosa* bacteraemia was associated with increased mortality, even though selection of antibiotics was based on clinical practice guidelines [57]. Thus, early administration of an appropriate targeted antibiotic is often desirable when the local epidemiology, susceptibility profile, and resistance mechanisms are known to guide antibiotic treatment. However, in the absence of such information, empiric therapy with the broadest coverage in terms of species and resistance mechanisms is warranted in hospital units with high rates of carbapenem resistance.

Among the antibiotics tested in this study, the highest predicted therapeutic coverage against key Gram-negative bacteria was found with colistin and, among the newer agents, cefiderocol. The data with cefiderocol supported previous in vitro susceptibility findings. Among isolates collected from Italian hospitals in the pre-COVID-19 era (2014–2018), overall susceptibilities to cefiderocol were 95.0%, 99.5%, and 88.1% for *A baumannii*, *P. aeruginosa*, and *K. pneumoniae*, respectively; the meropenem-resistant subsets of the same species had susceptibilities of 95.2%, 100%, and 67.9%, respectively, based on EUCAST breakpoints [58]. The cefiderocol susceptibility of 902 Italian Gram-negative isolates collected from patients with nosocomial pneumonia was approximately 97%, while susceptibility to comparator antibiotics ranged between 67% and 79% [58]. Similarly high susceptibilities to cefiderocol (97.7% for *A. baumannii* and > 99% for Enterobacterales and *P. aeruginosa*) were also reported for isolates collected in 2020 from hospitalised patients in the USA and in Europe [31]. The in vitro potency of cefiderocol extends to isolates with different phenotypic resistance profiles (e.g., isolates not susceptible to ceftolozane-tazobactam or ceftazidime-avibactam) and different molecular mechanisms [31–33]. Among the newer approved agents, cefiderocol has the broadest range of activity against Gram-negative species and various CR mechanisms, including Class A KPC, Guiana extended-spectrum (GES), extended-spectrum beta-lactamase (ESBL) enzymes, Class B metallo-beta-lactamases, Class D oxacillinases, and Class C chromosomal or acquired cephalosporinases [33, 59]. The high potency of cefiderocol against this wide variety of species and mechanisms can be linked to its structural stability against hydrolysis by the various types of beta-lactamases [60–62]. Such extensive coverage also contributes to the potential therapeutic utility of cefiderocol in ICUs as a form of empiric treatment when pathogen susceptibilities are unknown. Notably, the CREDIBLE-CR study has demonstrated the efficacy of cefiderocol in critically ill patients with infections due to CRE, CR *P. aeruginosa*, and CRAB isolates with the aforementioned resistance mechanisms; and mortality rates were similar to those reported in other studies [43, 45].

Following its approval in 2020, cefiderocol was utilised effectively during the pandemic in several Italian ICUs for the treatment of CRAB-associated VAP or BSI [21, 48, 49, 63]. In two of these studies – both observational, retrospective, cohort studies – cefiderocol was at least as effective as colistin in terms of all-cause mortality; for both treatments, sequential organ failure assessment (SOFA) score was an independent risk factor for mortality [21, 49]. According to the ARES observational study of hospitalised patients with COVID-19, cefiderocol treatment for those with secondary bacterial infections achieved good clinical efficacy, although overall in-hospital mortality rates remained very high [64]. In contrast, a recently published systematic review of four observational studies of patients with CRAB infections has found a reduced mortality risk with cefiderocol-based regimens compared with non-cefiderocol-based regimens after adjusting for confounders (odds ratio 0.53; 95% confidence intervals 0.39–0.71; *I*^2^ = 0.0%) [65].

Rapid diagnostics can help to identify the causative pathogen and its mechanism of resistance more rapidly than the conventional culture plus susceptibility testing, thereby allowing more timely treatments with the appropriate antibiotics that have proven in vitro activity [4, 27]. However, in the absence of accurate species and molecular information on the causative pathogens, antibiotic prescription should follow key principles related to local epidemiology, hospital, or ICU antibiogram, availability of the most active antibiotics, and antibiotic stewardship programmes (i.e., escalation or de-escalation, optimisation of plasma levels, shortening the treatment duration if feasible, oral stepdown) [27, 66]. A better understanding of clinical risk factors or biomarkers for disease progression in critically ill patients – such as multisite colonisation, mechanical ventilation, higher Charlson comorbidity index, continuous veno-venous haemofiltration, and C-reactive protein – may help to identify patients who could benefit the most from early appropriate treatment [67–69]. This is particularly important because challenging CR Gram-negative pathogens are often isolated from the same infection site in polymicrobial infections, thus highlighting the need for an agent with coverage against multiple pathogens [52].

There is flexibility within the Italian VAP guidelines to administer newer antibiotics as prolonged or continuous infusion in order to maintain target plasma levels [53]. For cefiderocol, however, the approved dosing recommendations do not include any guidance for higher doses or infusion durations that extend beyond 3 h. This is because the target attainment in the lung and plasma is likely to be achieved under the current dosing recommendations, including those for patient populations with moderate or severe renal impairment, renal replacement therapy, or augmented renal clearance [30, 70, 71].

The surveillance data used in this study correspond well with ECDC data showing stability over the past 10 years in national CR rates among WHO priority pathogens [6]. However, there were a number of limitations to the study. These included a lack of precise information regarding how many patients providing bacterial isolates for the surveillance programme were admitted to the ICU. It has been found that Gram-negative bacterial species collected in general wards may have lower resistance rates than isolates in the ICU [72]. The extrapolation was based on 624, a relatively low number of Gram-negative isolates, and additionally, not all antibiotics were tested across the different Gram-negative bacteria, precluding direct comparisons. There was also possible underestimation in antimicrobial resistance, given that isolates were collected during the first year of the COVID-19 pandemic, at a time when laboratories were conducting only partial surveillance for MDR Gram-negative bacteria.

## Conclusions

In conclusion, the current analysis suggests that among the preferred beta-lactam antibiotics, cefiderocol was the most active agent with the highest predicted therapeutic coverage and potential utility against Gram-negative pathogens collected in Italian ICUs. Thus, in settings where the species and antibiotic susceptibility are unknown, cefiderocol may be a promising treatment option when administered early for at-risk ICU patients with CR Gram-negative bacterial infections.

## Data Availability

No datasets were generated or analysed during the current study.

## Abbreviations

BSI: Bloodstream infection
CAMHB: Cation-adjusted Mueller-Hinton broth
CLSI: Clinical and Laboratory Standards Institute
COVID-19: Coronavirus disease 2019
CR: Carbapenem-resistant
CRAB: Carbapenem-resistant *Acinetobacter baumannii*
CRE: Carbapenem-resistant Enterobacterales
CRPA: Carbapenem-resistant *Pseudomonas aeruginosa*
DTR: Difficult-to-treat resistant
ECDC: European Centre for Disease Prevention and Control
ESBL: Extended-spectrum beta-lactamase
EUCAST: European Committee on Antimicrobial Susceptibility Testing
GES: Guiana extended-spectrum
GiViTI: Gruppo Italiano per la Valutazione degli Interventi in Terapia Intensiva
HAI: Hospital-acquired infection
HAP: Hospital-acquired pneumonia
ICU: Intensive care unit
ID-CAMHB: Iron-depleted cation-adjusted Mueller-Hinton broth
KPC: *Klebsiella pneumoniae* carbapenemase
MDR: Multidrug-resistant
MIC: Minimum inhibitory concentration
PDR: Pan-drug resistant
S: Susceptible
SOFA: Sequential organ failure assessment
UK: United Kingdom
USA: United States of America
UTI: Urinary tract infection
WHO: World Health Organization
VAP: Ventilator-associated pneumonia
XDR: Extensively drug-resistant
